# A new bedside prediction rule to assess liver allografts viability in icu admitted donors

**DOI:** 10.1186/2197-425X-3-S1-A895

**Published:** 2015-10-01

**Authors:** CR Hernández Socorro, R Prada Osorio, JL Romero Luján, V Peña Morant, C Sánchez Ramírez, A Aranzazu Anabirtarte, P Saavedra Santana

**Affiliations:** Las Palmas de Gran Canaria, Hospital Universitario de Gran Canaria Doctor Negrin, Spain; Las Palmas de Gran Canaria, Universidad de las Palmas de Gran Canaria, Spain

## Objectives

To determine a bedside prediction rule which unable us to predict liver allograft viability in order to optimize successful liver donation in potential liver donors admitted to ICU.

## Methods

A cross-sectional, single center study performed in a mixed ICU. Data from all the potential candidates for liver transplant donation in our center from January 2010 to November 2014 were analyzed. The studied variables were demographics, hypertension, hypotensive episodes, diabetes mellitus, dyslipidemia, infection on admission, cardiac arrest episodes, last glycemia, sodium, hemoglobin, APTT, urea, total proteins, total leukocytes, platelets, transaminases, gamma-glutamyl transferase, lactic dehydrogenase, alkaline phosphatase, antibiotic treatment and abdominal echography (7 categories) considering a positive sonographic study when it was: normal, ≤ 30% steatosis, cholecystitis or cholelithiasis or benign focal hepatic lesion. Categorical variables are expressed as frequencies and percentages, and continuous variables as mean and standard deviation (SD) when the data followed the normal distribution or medians and interquartile ranges (IQR) when they did not. The percentages were compared using the Chi-square test, the means by the t-test, and the medians by the Wilcoxon test for independent data. Those variables that showed in the univariate analyses with the outcome were introduced in a multivariate logistic analysis. A selection of variables based on the algorithm of complete enumeration and Bayesian information criterion (BIC). Models were summarized by a p-values odd-ratios, which were estimated by confidence intervals 95%. For the predictor deduced from the model, the corresponding ROC curve was obtained and the AUC was estimated by means of a 95 % CI. Statistical significance was set at p < .05. Data were analyzed using the R package

## Results

**Figure 1:**
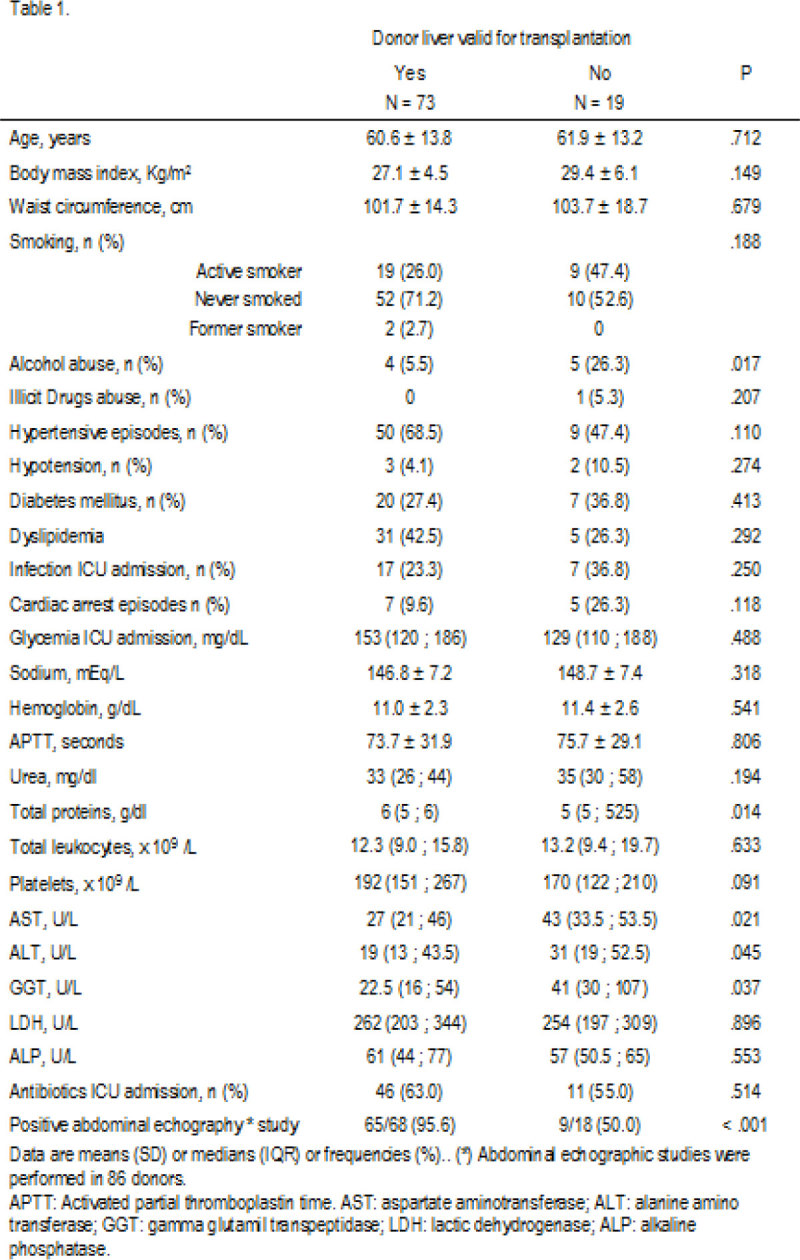
Figure 1

We assessed 93 candidates for liver transplant donation from brain-death donors, 74 of which were valid for transplant and 19 were discarded. Donors baseline characteristics are shown in Figure 1.

From the multiple logistic regression analysis the risk factors independently associated for liver allograft viability were platelets per unit Naperian logarithm OR (CI 95%): 8.158 (1.47; 45.27) p = .007, and positive abdominal ultrasound OR (CI 95%): 26.651 (5.19 ; 136.9) p < .001. The following prediction rule to assess liver allografts viability for transplant was obtained: Score: 2.0989 × NapLog (platelets) + 3.2828 × Echography. Echography takes the values 1 or 0, according if it is or not favorable to transplant. A liver is valid for transplant if score is greater than cut-off. For this score AUC was: 0.805 (95% CI: 0.678; 0.931). For a cut-off of 27.156 the predictor has a sensitivity of 94.1% and a specificity of 61.1%

## Conclusions

We have designed a new bedside score to predict liver allografts viability in ICU admitted donors of universal utilization.

